# Structural insights into the disulfide isomerase and chaperone activity of TrbB of the F plasmid type IV secretion system

**DOI:** 10.1016/j.crstbi.2024.100156

**Published:** 2024-07-14

**Authors:** Arnold J. Apostol, Nicholas J. Bragagnolo, Christina S. Rodriguez, Gerald F. Audette

**Affiliations:** a Department of Chemistry, York University, 4700 Keele St, Toronto, ON, Canada, M3J 1P3; b Centre for Research on Biomolecular Interactions, York University, Canada

**Keywords:** F-like type IV secretion system, Disulfide isomerase, Small angle X-Ray scattering, Circular dichroism spectroscopy, ^1^H–^15^N heteronuclear single quantum coherence spectroscopy, ColabFold-AlphaFold2, Protein design

## Abstract

Bacteria have evolved elaborate mechanisms to thrive in stressful environments. F-like plasmids in gram-negative bacteria encode for a multi-protein Type IV Secretion System (T4SS_F_) that is functional for bacterial proliferation and adaptation through the process of conjugation. The periplasmic protein TrbB is believed to have a stabilizing chaperone role in the T4SS_F_ assembly, with TrbB exhibiting disulfide isomerase (DI) activity. In the current report, we demonstrate that the deletion of the disordered N-terminus of TrbB_WT_, resulting in a truncation construct TrbB_37-161_, does not affect its catalytic *in vitro* activity compared to the wild-type protein (p = 0.76). Residues W37–K161, which include the active thioredoxin motif, are sufficient for DI activity. The N-terminus of TrbB_WT_ is disordered as indicated by a structural model of GST-TrbB_WT_ based on ColabFold-AlphaFold2 and Small Angle X-Ray Scattering data and ^1^H–^15^N Heteronuclear Single Quantum Correlation (HSQC) spectroscopy of the untagged protein. This disordered region likely contributes to the protein's dynamicity; removal of this region results in a more stable protein based on ^1^H–^15^N HSQC and Circular Dichroism Spectroscopies. Lastly, size exclusion chromatography analysis of TrbB_WT_ in the presence of TraW, a T4SS_F_ assembly protein predicted to interact with TrbB_WT_, does not support the inference of a stable complex forming *in vitro*. This work advances our understanding of TrbB's structure and function, explores the role of structural disorder in protein dynamics in the context of a T4SS_F_ accessory protein, and highlights the importance of redox-assisted protein folding in the T4SS_F_.

## Highlights

• TrbB of the F-plasmid Type IV Secretion System functions as a disulfide isomerase.
• Predicted models of TrbB highlight regions of increased flexibility.
• Structurally, TrbB has a flexible N-terminus and C-terminal thioredoxin domain.
• Truncation of TrbB increases stability and facilitates structural analysis.
• The C-terminal region of TrbB is sufficient for disulfide isomerase activity.

## Graphical abstract

**Figure:**
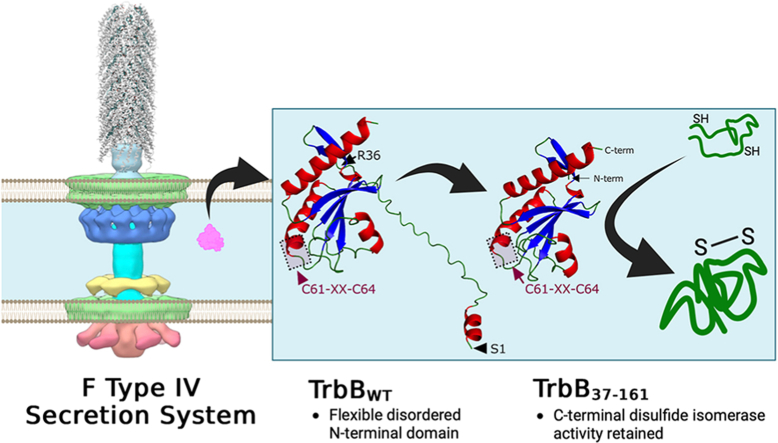

## 1 Introduction

Bacteria utilize a multitude of mechanisms to survive in stressful and competitive environments, including mechanisms that can harm other cells. Secretion systems (SSs) are large multi-protein complexes expressed by bacteria that aid in their survival due to their numerous functionalities (Costa et al., 2015; Filloux, 2022; Green and Mecsas, 2016; Nicholson and Champion, 2022). SSs can breach a target cell's lipid membrane(s), allowing them to perform acts of host pathogenesis by mediating the secretion of toxins and virulence genes, enhancing bacterial survivability, attaching to host cells, eliminating target cells and thus outcompeting the host cell for resources in the immediate environmental niche. There are a total of eleven types of multi-protein SSs found in the bacterial kingdom, some of which are categorized into subtypes and each classified based on the pathway of secretion and the type of secreted macromolecule (Costa et al., 2015; Trivedi et al., 2022). Some SSs, such as Type III and Type VI, mediate inter-microbial competition through the injection of effector proteins from the cytoplasm directly across host membranes (Allsopp et al., 2020; Cianfanelli et al., 2016; Trivedi et al., 2022). Other SSs, such as Type IV SS, mediate the export of both proteins and DNA from the cytosol of T4SS-containing bacteria to either a prokaryotic or a eukaryotic host cell, and it is central in the import of extracellular DNA or a toxic protein (Beck et al., 2014; Bragagnolo et al., 2020; Grace et al., 2019; Trivedi et al., 2022). In gram-negative bacteria bearing F-like plasmids, the Type IV SS (T4SS_F_) is functional for bacterial proliferation and adaptation through the process of conjugation, a process that is also clinically relevant as it arbitrates the spread of antibiotic resistance genes among bacteria.

Ubiquitous to SSs are disulfide isomerases (DIs) and protein-folding-assisting pathways that ensure the structural and physicochemical stability of substrate proteins through the re-arrangement of intramolecular disulfide bonds (Karimi et al., 2016; Zavodszky et al., 2001). In gram-negative bacteria, redox-assisted protein folding occurs mainly in the periplasm, due to its higher reduction potential of −165 mV (Messens et al., 2007) compared to that of the cytoplasm, between −260 and −280 mV (Hoober et al., 1999; Hwang et al., 1992; Silhavy et al., 2010). Interestingly, disulfide-bond-forming pathways in gram-negative bacteria are believed to be non-essential for growth (Bardwell et al., 1991), yet they are required for pathogenesis (Heras et al., 2009).

In gram-negative T4SSs encoded by F-like plasmids (F, R100, pSLT, pED208, and pYJ016), the *trbB* and *traF* genes are two of six genes that have no orthologues in P- or I-like subfamilies, and each protein contains a thioredoxin motif, a structural domain consisting of a minimum of three α-helices flanking a four-stranded antiparallel β-sheet (Bragagnolo et al., 2020; Collet and Bardwell, 2002; Elton et al., 2005; Frost et al., 1994; Kinch et al., 2003; Lawley et al., 2003; Missiakas and Raina, 1997). The TraF thioredoxin-like domain does not contain the characteristic CXXC active site, and TraF's function in T4SS_F_ pilus extension, independent of redox activity, remains unclear. TrbB contains an active thioredoxin domain with a CXXC active site (Audette et al., 2004; Collet and Bardwell, 2002; Elton et al., 2005; Frost et al., 1994).

TrbB has been shown to function as a DI *in vitro* and *in vivo* (Elton et al., 2005; Frost et al., 1994; Hemmis et al., 2011; Lawley et al., 2003), suggesting that TrbB may ensure proper protein folding of T4SS_F_ proteins. Several T4SS_F_ proteins are remarkable for their high cysteine content and these proteins are putative client proteins for TrbB; TraN, TraU, and TraH each have 22, 11, and 6 cysteines, respectively (Bragagnolo et al., 2020; Frost et al., 1994; Hemmis et al., 2011). Additionally, TraW, another T4SS_F_ protein functional in pilus assembly (Bragagnolo et al., 2020; Maneewannakul and Maneewannakul, 1992), was previously identified as a binding partner for TrbB (Harris and Silverman, 2004). Conjugative T4SSs express TrbB alone, or TraF/TrbB; non-redox active TraF alone is not observed (Bragagnolo et al., 2020; Elton et al., 2005). For instance, the F and pED208 plasmids contain both TraF and TrbB while the R27 has only a redox active TraF, also suggesting different roles for TraF and TrbB within the T4SS_F_ conjugative apparatus. Here, we report solution-based biophysical and computational structural analyses, advancing our understanding of TrbB's structure, enzymatic activity, and its interaction with other T4SS_F_ proteins.

## 2 Materials and methods

### 2.1 Cloning, protein expression and purification

Cloning of *trbB*_*37-161*_ into the vector pGEX-4T-2 was done by PCR amplification from the T4SS_F_-containing pOX38 plasmid (Anthony et al., 1994). The forward primer used was 5′-GTACTGAATTCaatggttccgtctcagtaatggcagg-3’; the reverse primer used was 5′-GTCTTGTCGACttatttcgcaccttttttcctccgtacatctgc-3’ (Integrated DNA Technologies). Amplicon and vector were double digested with *Eco*RI and *Sal*I, ligated *in vitro*, and constructs were transformed into *Escherichia coli* by heat shock. Cloning was verified by colony PCR, mini-prepping plasmids from colonies followed by double-digestion with restriction enzymes, and by Sanger sequencing at The Centre for Applied Genomics (The Hospital for Sick Children, Toronto, ON) using standard pGEX primers supplied by the institution. Other expression plasmids, encoding for GST-TrbB_WT_ (aa 21–161), His_6_TraW, and His_6_ΔTraW (aa 51–210) were from lab stocks.

Proteins were expressed from *E. coli* BL21(DE3) cells transformed with or bearing the construct DNA of interest, and were incubated at 37 °C in Luria-Bertani broth supplemented with 50 μg/mL ampicillin to mid-log growth (OD_600_∼0.4–0.7) at 37 °C. Large-scale expression was induced using 1 mM Isopropyl β-D-1-thiogalactopyranoside (IPTG) overnight at 18 °C. Cells were separated from the liquid growth media by centrifugation (5000×*g*, 40 min, 4 °C), re-suspended in buffer (50 mM Tris pH 7.5, 150 mM NaCl, 1 mM EDTA) supplemented with protease inhibitors (1.25 mM phenylmethylsulfonyl fluoride, 5 mM benzamidine HCl, 5 mM aminocaproic acid), and sonicated at 25% amplitude for 5 min (on: 10s, off: 30s). Soluble cell content was separated from insoluble cellular debris by centrifugation (25000×*g*, 40 min, 4 °C) and tagged proteins were isolated using GST-Sepharose or Ni^2+^-NTA column chromatography on an ÄKTA Purifier 10S FPLC system (Cytiva). Tagged proteins were eluted with 20 mM GSH or 500 mM imidazole as appropriate for the particular fusion tag; protein purity was ascertained through SDS-PAGE. Pooled protein was quantified using UV–Vis spectroscopy at A_280_ (extinction coefficients: GST-TrbB_WT_ 65695 M^−1^cm^−1^; TrbB_WT_ 22585 M^−1^cm^−1^; TrbB_37-161_ 17085 M^−1^cm^−1^; His_6_TraW 22460 M^−1^cm^−1^; His_6_ΔTraW 11460 M^−1^cm^−1^ [determined using the Edelhoch method; Edelhoch, 1967; Gill and von Hippel, 1989; Pace et al., 1995]) or by bicinchoninic acid (BCA) assay (Pierce, Thermo Fisher), and concentrated using 10 kDa or 30 kDa MWCO Amicon centrifugal concentrators (Millipore-Sigma). GST-TrbB_WT_ and GST-(TrbB)_37-161_ were liberated from their GST-tag by incubation with Thrombin (5–10 units/mg of GST-tagged protein expressed from a pGEX-4T-2 construct; Millipore-Sigma) or HRV-3C Protease (2 units/0.2 mg of GST-tagged protein expressed from a pGEX-6P-2 construct; Pierce, Thermo Fisher) overnight at 4 °C in 1X phosphate-buffered saline (PBS) supplemented with 1 mM dithiothreitol (DTT). Secondary purification using Size-Exclusion Chromatography (SEC) was also performed using HiPrep 16/60 Sephacryl S-100 HR column on an ÄKTA Purifier 10S FPLC system (Cytiva) to isolate the untagged protein.

### 2.2 Circular Dichroism (CD) spectroscopy

CD spectra were acquired between 200 and 260 nm using a Jasco J810 CD spectrometer at 22 °C and a protein concentration of 5 μM in 10 mM KH_2_PO_4_ pH 7.5, 100 mM kF, 5% (v/v) glycerol to minimize noise (Greenfield, 2007). Following acquisition, CD spectra were input into BeStSel (Micsonai et al., 2022) for secondary structure content prediction. Parameters include units of measured ellipticity (mdeg) at 5 μM, the number of residues (TrbB_37-161_: 125, TrbB_WT_: 161), and the pathlength (0.1 cm). Thermal denaturation measurements were performed by sampling from a single wavelength (222 nm for α-helices) as a function of temperature (30–90 °C) at a rate of 1 °C/min. All CD experiments were performed in triplicate, and the mean of the internal replicates are reported. Thermal denaturation spectra data points are mean of three internal replicates with the standard deviation represented as error bars.

### 2.3 ^1^H–^15^N Heteronuclear Single Quantum Correlation (HSQC) Nuclear Magnetic Resonance (NMR) spectroscopy

TrbB_37-161_ and TrbB_WT_ were expressed from *E. coli* BL21(DE3) grown in M9 minimal media (6 g of Na_2_HPO_4_, 3 g of KH_2_PO_4_, 1 g of ^15^NH_4_Cl, 0.5 g of NaCl, and 10 g of glucose in 1 L of water supplemented with 1 mM CaCl_2_, 1 mM MgSO_4_, 50 μg/mL kanamycin, and a trace mineral mix). Large-scale protein expression and purification was done as described in Section 2.1. Purified protein samples were concentrated to 0.2 mM and supplemented with 10% (v/v) D_2_O in 10 mM HEPES pH 6.0, 50 mM NaCl. HSQC spectra were acquired on a Bruker DRX 600 NMR spectrometer operating at a ^1^H frequency of 599.80 MHz at 21 °C.

### 2.4 SEC-MALS small angle X-ray scattering

Size exclusion chromatography linked to multi-angle light scattering and small angle X-Ray scattering (SEC-MALS-SAXS) data for GST-TrbB_WT_ was collected at the BioCAT 18ID beamline (Advanced Photon Source, Argonne National Laboratory, USA). The protein sample was prepared without GST cleavage as per Section 2.1 and was buffer exchanged into a 10X dilution of a buffer stock (20 mM HEPES pH 7.0, 100 mM NaCl, 5% [v/v] glycerol, 0.05% NP40) using a 30 kDa MWCO concentrator. The 5.5 mg/mL GST-TrbB_WT_ sample (as quantified via the Edelhoch method; Edelhoch, 1967; Gill and von Hippel, 1989; Pace et al., 1995) and the matching buffer were sent to the BioCAT facility where the buffer was diluted 10X and used as the running buffer for the SEC-MALS-SAXS experiment. GST-TrbB_WT_ was injected at a volume of 350 μL onto a Superdex 200 10/300 Increase SEC column (Cytiva) using an Agilent Infinity II HPLC at 0.6 mL/min, and the sample underwent sequential multi-angle laser light scattering analysis using a Wyatt DAWN Heleos II MALS system; protein concentration was determined in-line with a refractive index detector. SAXS data was acquired using an Eiger2 XE 9M detector at a sample-detector distance of 3.7 m and at λ = 0.1033 nm. Data processing was performed using BioXTAS RAW v.2.1.1 and ATSAS packages (Hopkins et al., 2017; Manalastas-Cantos et al., 2021). *Ab initio* reconstruction was performed using DAMMIF (30 independent runs) with DAMAVER averaging and refinement using the ColabFold-AlphaFold2 (CF-AF2) GST-TrbB_WT_ model, and clustering using DAMCLUST. Subsequently, the Ensemble Optimization Method (EOM) was employed to gain further insight into the structural disorder of the protein system (Bernadó et al., 2007; Tria et al., 2015). The experimental SAXS data was utilized to generate a pool of predicted models in various conformations from which a selected representative ensemble was used to determine the protein's degree of conformational polydispersity. The program was accessed through ATSAS Online (Gajoe version 2.1) (Petoukhov et al., 2007) where EOM settings were configured to 20 maximum number of curves per ensemble, 5 minimum number of curves per ensemble, with constant subtraction and curve repetition in the ensemble allowed, 100 cycles of the genetic algorithm run, and 10,000 theoretical curves. The assigned protein parameters that provided the best model were when the protein was designated as monomer with residues 1–218 as compact, 219–271 as disordered, and 272–396 as compact. The program CORAL (complexes with random loops) was also employed via ATSAS online to determine whether the SAXS data better fits a monomeric or dimeric construct by validating models of input rigid bodies with constrained positionalities connected by a generated disordered linker (Petoukhov et al., 2012). Models of the GST monomer and the TrbB_37-161_ monomer from CF-AF2 were used as the input for the domains in modeling the GST-TrbB monomer with CORAL; a linker of 46 residues (10 disordered residues belonged to the GST tag) was specified between the C-terminal of the GST model and the N-terminal of the TrbB_37-161_ model. Angular units were set to 1/Å based on the input SAXS data and symmetry was set to P1. In modeling the GST-TrbB dimer, multiple CORAL experiments were performed where two copies of each model with the linker were specified either with no contacts, or with contact conditions and domain groupings for a GST dimeric interface, a TrbB_37-161_ dimeric interface, and both GST and TrbB_37-161_ interfaces. Bead models were visualized using PyMOL v2.5.2 (Schrödinger Inc.), where the bead radius was set to 1.5 for TrbB's N-term and 5.4 for the rest (based on the size set by DAMMIF). CF-AF2 predicted models were also aligned with the SAXS-EOM bead models using PyMOL v.2.5.2. SAXS data for GST-TrbB_WT_ were deposited to the SASBDB (https://www.sasbdb.org); accession code: SASDTL5.

### 2.5 Fluorometric disulfide isomerase *in vitro* assay

PDI activity was assessed using a fluorometric protein disulfide isomerase quenched-fluorophore kit from Abcam (Boston, USA; cat ab273337). TrbB_WT_ and TrbB_37-161_ were buffer exchanged into the kit's PDI assay buffer supplied by the manufacturer prior to analyses. Signal detection was performed using a Synergy H4 Microplate reader (Agilent BioTek) with excitation and emission wavelengths of 490 nm and 580 nm, respectively, at 25 °C. Data were collected in triplicate (n = 3) from two independently expressed and purified protein samples with final concentrations 50 μM in each well. Statistical t-tests were performed using GraphPad Prism v.9.5.1 (Dotmatics).

### 2.6 Analytical UPLC Size-Exclusion Chromatography

TrbB_WT_, TrbB_37-161_, His_6_TraW, and His_6_ΔTraW at 19 μM in 20 mM HEPES pH 7.5, 150 mM NaCl, with or without 2 mM DTT, were analyzed using a Zenix SEC-150 column (Sepax Tech. Inc.) at a rate of 1.0 mL/min on an H-class Ultra Performance Liquid Chromatography (UPLC) system (Waters Acquity) with A_280_ sample detection, and sample peaks were compared to a Gel Filtration Standard of proteins with known MW and elution profiles (Bio-Rad; No. 1511901) to qualitatively gauge sample size and oligomeric state. The column was washed with 1 column volume of buffer (15 mL of 20 mM HEPES pH 7.5, 150 mM NaCl) prior to every protein analysis. The TrbB_WT_/His_6_TraW, TrbB_WT_/His_6_ΔTraW, TrbB_37-161_/His_6_TraW mixtures were equilibrated on ice for at least 30 min prior to SEC analysis to allow for any complex formation. TrbB_WT_/His_6_ΔTraW was analyzed to determine whether the deletion of the flexible N-terminus of TraW would affect the observation of a complex. Samples were also reduced in 2 mM Dithiothreitol (DTT), including TrbB, and analyzed with SEC.

### 2.7 Structural modelling of TrbB

Computational 3D protein structure models were generated using the primary sequence of the relevant protein as input into CF-AF2 (Mirdita et al., 2022) site (https://colab.research.google.com/github/sokrypton/ColabFold/blob/main/AlphaFold2.ipynb) using default settings. These default settings include MSA mode (set to mmseqs2_uniref_env), pair mode (set to unpaired_paired), model type (set to alphafold2_ptm or alphafold2_multimer_v3, used to model heterodimeric protein binding), number of recycles was set to 3, recycle_early_stop_tolerance (auto), pairing strategy (greedy); sample settings included max msa (auto) and number of seeds (1). Predicted structure models were visualized in PyMOL v2.5.2 (Schrödinger Inc.) and aligned with the SAXS-EOM bead models. Reported predicted Template Modelling (pTM) metric (range ∈ [0,1]) indicates CF-AF2's confidence in its predicted model's reliability. Indicated confidence metric, in the range ∈ [0,1], is given by 0.8*ipTM + 0.2*pTM, preferably weighing the metric for the reliability of binding interfaces (interface predicted Template Modelling score, ipTM) rather than the pTM score (Evans et al., 2021).

## 3 Results and discission

### 3.1 TrbB_WT_ and TrbB_37-161_ are physicochemically distinct

**Fig. 1:**
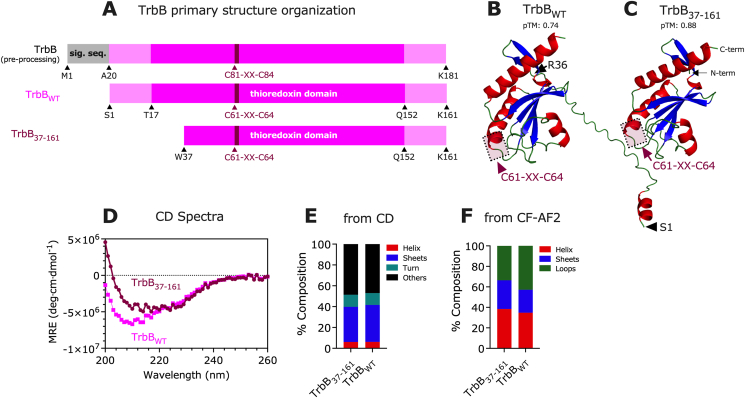
**Structural organization of TrbB**_**WT**_**and TrbB**_**37-161**_. (A) Domain structures based on primary sequence of pre-processed TrbB (signal sequence intact), TrbB_WT_ (following removal of the signal sequence), and the truncation construct TrbB_37-161_. ColabFold-AlphaFold2 predicted 3D models of (B) TrbB_WT_ and (C) TrbB_37-161_. (D) Circular Dichroism spectra expressed in mean residue ellipticity (MRE) of TrbB_WT_ (magenta) and TrbB_37-161_ (maroon) were (E) analyzed using BeStSel (Micsonai et al., 2022) to indicate secondary structure composition. CD measurements were collected in triplicate from 5 μM protein samples at 22 °C. For comparison to the empirical data, (F) CF-AF2 secondary structure composition was determined by normalizing number of residues predicted to form α-helices, β-sheets, or loops, by the total number of residues in the protein construct.

**Fig. 2:**
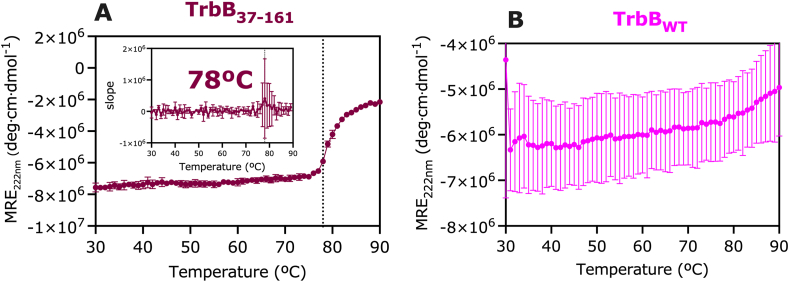
**Thermal denaturation profiles of** α**-helices in (A) TrbB**_**37-161**_**and (B) TrbB**_**WT**_. Circular dichroism at 222 nm expressed in mean residue ellipticity (MRE) of 5 μM protein samples was monitored as a function of temperature. The midpoint of unfolding (T_M_) is given by the temperature at which the first derivative of the CD vs T curve is at its highest; T_M_ of TrbB_37-161_ is 78 °C (in all replicates), that of TrbB_WT_ cannot be reliably ascertained due to significant data deviations. Data points are shown as mean ± SD, n = 3 internal replicates.

In the context of the T4SS_F_, many component proteins have highly dynamic regions (Bragagnolo et al., 2020; Bragagnolo and Audette, 2022). Wild-type TrbB (TrbB_WT_) was predicted by ColabFold-AlphaFold2 (CF-AF2) to have a disordered N-terminal domain (Fig. 1A, B; Supplementary Fig. S1). This disordered region comprises about a quarter of the wild-type protein (Fig. 1A), contributes to its flexibility, and likely plays a role in its interaction with multiple substrate proteins as a disulfide isomerase (DI) (Arhar et al., 2021). The TrbB_37-161_ construct was designed to remove this disordered region (Fig. 1A-C); whether this region is involved in substrate binding is unclear, though it is not required for disulfide isomerase activity (Section 3.3), and the truncated protein is a more stable construct. CD spectroscopy of TrbB_WT_ and TrbB_37-161_ indicate modest changes at the secondary structure level between the wild-type and truncated forms (Fig. 1D). The percentage of α-helical content incrementally decreases from 6.2% in TrbB_WT_ to 6.1% TrbB_37-161_ (Fig. 1E). β-Sheet composition modestly decreases from 35.4% in TrbB_WT_ to 33.7% TrbB_37-161_, respectively (Fig. 1E). The thermal denaturation of TrbB_37-161_ indicates that, while it is comprised of only 6.1% α-helix, it is a stable construct with a T_M_ of 78 °C (Fig. 2A). The predicted model (Fig. 1C) organizes the α-helices in an amphipathic fashion, with 43.8% of helix-forming residues being polar (25.0%) or charged (18.7%) (Supplementary Fig. S2), fostering a stable packing of the helices within the thioredoxin-like motif. Conversely, the thermal denaturation profile of TrbB_WT_ is strikingly different from TrbB_37-161_ and inconsistent across replicates (Fig. 2B), making it difficult to ascertain its T_M_ precisely. Thus, while the two constructs have very similar secondary structures, only TrbB_37-161_ is thermally stable. We infer that the wild-type protein fails to have a consistent thermal denaturation profile because of the generally disordered and dynamic N-terminal region, making it less stable and more susceptible to aggregation.

**Fig. 3:**
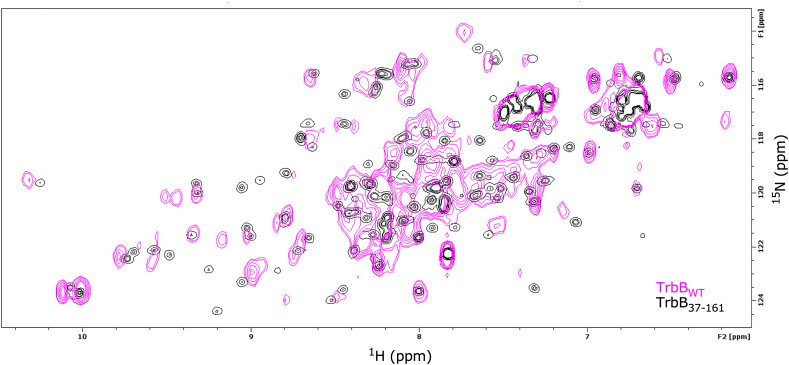
**N-terminal truncation of TrbB leads to a more stable protein.** Shown is an overlap of the ^1^H–^15^N Heteronuclear Single Quantum Correlation (HSQC) spectra of TrbB_WT_ (magenta) and TrbB_37-161_ (black). Protein samples, at a concentration of 0.2 mM in 10 mM MES pH 6.0, 50 mM NaCl, 10% (v/v) D_2_O, were analyzed at 600 MHz. The increase in well resolved peaks in the TrbB_37-161_ spectrum indicates a less structurally dynamic and more stably folded protein.

TrbB_WT_ and TrbB_37-161_ were characterized with ^1^H–^15^N Heteronuclear Single Quantum Correlation (HSQC) Nuclear Magnetic Resonance (NMR) spectroscopy (Fig. 3). A significant distinction between TrbB_WT_ and TrbB_37-161_ was observed; the signals arising from the wild-type protein are evidently less resolved whereas the signals from the truncation mutant are smaller, concentric, and more resolved. Peak assignment and structure solution is more feasible for TrbB_37-161_ due to its resolved peaks (Fig. 3). Two inferences can be drawn from the signals between 8 and 8.5 ppm in the ^1^H dimension (x-axis). First, TrbB_WT_ (magenta) is partially disordered, as the clustering of signals in this region corresponds to partial disorder in a protein (Kwan et al., 2011; Marion, 2013). Removal of the N-terminus in TrbB_37-161_ (black) results in better peak resolution, indicative of a protein with a more well-defined fold.

The signal differences between the two samples indicate that the removal of the N-terminal region resulted in a thermally and structurally stable TrbB_37-161_ construct. While the two constructs have very similar secondary structures (according to CD, Fig. 1D and E), the NMR of TrbB_WT_ is distinct from TrbB_37-161_ (Fig. 3) because the presence or absence of the N-terminus creates distinct intramolecular environments. Together, the similar secondary structures indicated by CD and the distinct NMR of TrbB_WT_ versus TrbB_37-161_ suggest that the N-terminus does not affect the protein's secondary structures, but its flexibility and dynamicity allow it to occupy different locales of the protein in a transient manner. The transient spatial proximity of the N-terminus to a particular locale of the protein at a given moment alters the magnetic environment of that part of the protein, resulting in the observed cross-peak overlaps (Fig. 3, magenta) (Forsberg et al., 2023; Schiavina et al., 2024). Overall, given that thermally and structurally stable proteins are generally more amenable to crystallization (Derewenda, 2004; McPherson and Gavira, 2014), TrbB_37-161_ is a better construct for ongoing crystallization efforts.

We investigated whether there are differences between empirical and computational methods with respect to estimating secondary structures for TrbB_WT_. Comparing computational data from CF-AF2 and empirical data from CD spectroscopy, differences in percentage composition between the two methods are evident (Fig. 1E, **F**). The CF-AF2 predicted model of TrbB_WT_ indicates a secondary structure composition of 34.8% α-helix and 22.40% β-sheet (Fig. 1F). In contrast, CD spectroscopy empirically determined the secondary structure content at 6.2% α-helix and 35.4% β-sheet (Fig. 1E). In the TrbB_37-161_ truncation mutant, CF-AF2 predicts protein composition of 38.4% α-helix and 28.0% β-sheet (Fig. 1F); CD spectroscopy indicates 6.1% α-helix and 33.7% β-sheet (Fig. 1E). Comparing the CF-AF2 predicted models of the wild-type protein (Fig. 1B) and the truncated construct (Fig. 1C) shows the removal of the predominantly disordered region (Supplementary Fig. S1, dotted circle). Therefore, the increase in composition of α-helix and β-sheet in TrbB_37-161_ can be attributed to the decrease in overall size of the protein, given that the percentage composition shown in Fig. 1F was calculated by normalizing the number of residues predicted to form α-helices or β-sheets by the total sequence of the protein. Differences between CF-AF2 and empirical CD data highlight the importance of utilizing a diverse set of tools in a post-AlphaFold era for efficient hypotheses-testing and problem solving (Terwilliger et al., 2023).

Intrinsically disordered and/or highly dynamic regions are increasingly becoming appreciated as functional moieties in proteins, highlighted by discoveries of their function in interactomes (Norris et al., 2023; Schlessinger et al., 2007). These disorder-based interactions are gaining more attention and are being found to transiently bind a diverse set of partner proteins at high specificity (Sharma et al., 2015; Uversky, 2011; Wright and Dyson, 2015). Furthermore, these proteins tend to self-associate and form stable aggregates (Uversky, 2010). Interestingly, the formation of stable protein aggregates, such as the accumulation of aggregated Tau in neuronal cells, is clinically relevant as it is a hallmark of Alzheimer's Disease (Irvine et al., 2008; Mochizuki et al., 2018; Trejo-Lopez et al., 2022). Lastly, comparative studies have shown that intrinsically disordered proteins (IDPs) or regions (IDRs) generally have low hydrophobicity and exhibit large net charge at physiological pH (Babu, 2016; Uversky et al., 2000). Based on CF-AF2, we hypothesized that the N-terminal region of TrbB_WT_, which is about a quarter of TrbB_WT_ (Fig. 1A), behaves as a disordered region. The high isoelectric point (pI 10.7) of the N-terminal region (S1-R36), suggests that it has a large net positive charge at physiological pH, supporting the proposal of its intrinsic disorder (Babu, 2016; Uversky et al., 2000). This hypothesis can explain why TrbB_WT_ forms stable aggregates at concentrations above 5 mg/mL, making it difficult to study using methods that require a considerable protein concentration such as SEC-MALS-SAXS or X-Ray crystallography.

### 3.2 TrbB_WT_ is highly dynamic partly due to its disordered N-terminus

TrbB_WT_ was further analyzed using SEC-MALS-SAXS. TrbB_WT_ is highly prone to aggregation at concentrations above 5 mg/mL, so the GST-tagged construct was utilized for this experiment to increase its solubility, making it amenable to SEC-MALS-SAXS analysis. Following SAXS data reduction, Guinier, Kratky, and Pair-distance [P(r)] distribution analyses were performed, analyses that indicated a flexible and dynamic GST-TrbB_WT_. Consequently, the Ensemble Optimization Method (EOM) was employed to obtain a better understanding of the protein's structural flexibility and dynamics (Da Vela and Svergun, 2020; Tossavainen et al., 2018; Tria et al., 2015).

**Fig. 4:**
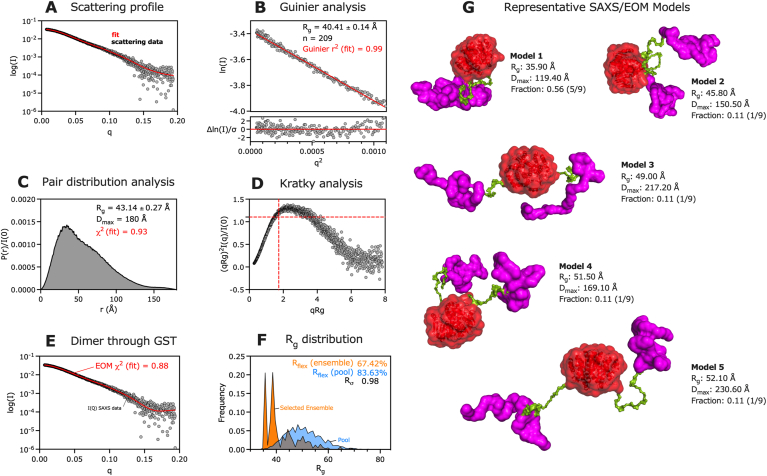
**SEC-MALS-SAXS analysis of TrbB**_**WT**_**indicates a structurally dynamic protein**. (A) SAXS scattering profile, (B) Guinier fit analysis, (C) Pair-distance [P(r)] distribution analysis from GNOM, and (D) Kratky analysis of 5.5 mg/ml GST-TrbB_WT_. (E) The Ensemble Optimization Method (EOM) was performed, with the goodness-of-fit metric displayed, to further analyze the structural dynamics of the protein. (F) The EOM-generated distribution of the randomly generated conformations (pool, blue curve) and the selected ensemble that fit the SAXS data (orange curve) are shown, along with metrics of conformational disorder (*R*_*flex*_, *R*_*σ*_). (G) Models from EOM-SAXS analysis showing GST-TrbB_WT_'s conformational polydispersity, colour-coded to emphasize the GST moiety (red), the disordered N-terminus of TrbB_WT_ (green), and TrbB_37-161_ (magenta). (For interpretation of the references to colour in this figure legend, the reader is referred to the Web version of this article.)

SAXS provides structural information from signal-averaged X-ray scattering of the protein as it freely diffuses, interacts with solvent molecules, and occupies 3D space in solution (Fig. 4A), meaning the dynamics of the protein in solution can be inferred from the data (Jacques and Trewhella, 2010; Putnam et al., 2007). The reliability of the collected SAXS data is backed by well-fit Guinier (Fig. 4B, *r*^*2*^ = 0.99) and P(r) distribution (Fig. 4C, *χ*^*2*^ = 0.93). Further, the radius of gyration (R_g_) values from the Guinier (40.41 ± 0.14 Å) and P(r) from GNOM (43.14 ± 0.27 Å) analyses are within an agreeable range (Table 1). The R_g_ is the weighted root mean square of the intramolecular distances with respect to the centroid of the electron density, effectively quantitating the size of the macromolecule in solution (Da Vela and Svergun, 2020). In other words, R_g_ is a radial averaging of all the conformations sampled by the macromolecule in solution.

**Table 1:** SEC-MALS-SAXS parameters for GST-TrbB_WT_.

Data collection parameters		
Instrument	BioCAT (Sector 18, APS)	
Detector	Eiger2 XE 9M	
Wavelength (Å)	1.033	
q-measurement range (1/Å)	0.0028 to 0.42	
Exposure time (s)	0.5	
Size exclusion column	Superdex 200 10/300 Increase	
Flow rate (mL/min)	0.6	
Temperature (°C)	20	
Protein concentration (mg/mL)	5.5	
Loaded volume (*μ*L)	300	
Buffer	20 mM HEPES pH 7.0, 100 mM NaCl, 5% glycerol, 0.05% NP40	
**Structural parameters**		
	**P(r) from GNOM**	**Guinier**
I(0)	0.0348 ± 1.15 × 10^−4^	0.0342 ± 7.41 × 10^−5^
R_g_ (Å)	43.14 ± 0.27	40.41 ± 0.14
D_max_ (Å)	180	

The observed molecular weight (MW) from SEC-MALS (86.0 kDa; Supplementary Fig. S3) and SAXS (Vp: 119.9 kDa; Vc: 102.6 kDa; Shape and Size: 106.4 kDa; and Bayes: 109.1 kDa) are over-estimated compared to the theoretical MW of a monomeric GST-TrbB_WT_ (46.4 kDa; Supplementary Fig. S3). The experimental MW values are closer to the theoretical MW value of a homodimer (92.8 kDa). The differential refractive index (dRI) trace (blue) from SEC-MALS does not suggest the presence of large protein aggregates; instead, it indicates the elution of a homogenous fraction (Graewert et al., 2020; Some et al., 2019). The MW estimate (red) across the dRI peak indicates the presence of a dimeric construct (86.0 kDa, Supplementary Fig. S3).

To supplement the SEC-MALS data, CORAL (Petoukhov et al., 2012) was employed to determine whether a monomeric (Supplementary Fig. S4A) or dimeric construct (Supplementary Figs. S4B–D) better fits the GST-TrbB_WT_ SAXS profile. Results indicate that a monomeric construct has a poorer fit (χ^2^ = 14.66, Supplementary Fig. S4A) compared to a dimeric construct that interacts through GST molecules (χ^2^ = 4.72, Supplementary Fig. S4B) or through TrbB_37-161_ molecules (χ^2^ = 1.41, Supplementary Fig. S4C). The fit becomes poorer when a construct where both self-dimerized GST and TrbB_37-161_ is considered (χ^2^ = 15.77, Supplementary Fig. S4D). Interestingly, a dimer through TrbB_37-161_ fits the SAXS data better compared to a dimer through GST (χ^2^ = 1.41 [Supplementary Fig. S4C] and χ^2^ = 4.72 [Supplementary Fig. S4B], respectively), suggesting that the observed construct from SEC-MALS-SAXS is a GST-TrbB_WT_ homodimer linked through their TrbB_37-161_ moieties. On one hand, the presence of C61 and C64 residues in its primary sequence (Fig. 1A; Supplementary Fig. S2A) leaves room for the possibility that it can dimerize through intermolecular disulfide bonds (Gavshina et al., 2021; Khanal et al., 2018; Rotoli et al., 2018), but it is unlikely that the active thioredoxin motif is involved in forming intermolecular linkages. Further, previous sedimentation experiments by Hemmis and colleagues (2011) have established that TrbB is monomeric in solution. The improved fit is likely due to the transient folding and unfolding of TrbB_37-161_, allowing it to pack with itself better transiently. On the other hand, the GST protein is known to dimerize through disulfide bridges, forming stable homodimers (Bell et al., 2013; Fabrini et al., 2009; Maru et al., 1996). This dimerization process can partly explain the over-estimated experimental molecular weights (Supplementary Fig. S3). Therefore, the observed over-estimated MWs strongly support a homodimeric GST-TrbB_WT_ (Da Vela and Svergun, 2020; Hopkins et al., 2017; Jacques and Trewhella, 2010; Putnam et al., 2007), likely linked through their GST moieties.

Based on the CF-AF2 predicted model, we hypothesized that the over-estimated MWs were a result of the protein's flexibility and dynamicity. TrbB_WT_'s dynamic N-terminal region (Fig. 1A-**B**) increases the observed R_g_ as it extends away from the centroid of the protein (Supplementary Fig. S4, diagram). The monomeric protein Ovalbumin, whose MW (42.5 kDa) is similar to monomeric GST-TrbB_WT_ (46.4 kDa), has an R_g_ value (23.1 Å) about 50% lower than that of GST-TrbB_WT_ (40.4 Å by Guinier, Fig. 4B; and 43.1 Å by P(r) from GNOM, Fig. 4C and Table 1) (Smilgies and Folta-Stogniew, 2015). Homodimeric proteins within a similar MW range as homodimeric GST-TrbB_WT_ (SEC-MALS: 86.0 kDa; Vp: 119.9 kDa; Vc: 102.6; Shape and Size: 106.4 kDa; and Bayes: 109.1 kDa; Supplementary Fig. S3) have R_g_ values that are also ∼50% lower compared to GST-TrbB_WT_ (homodimeric yeast enolase, MW: 79.5 kDa, R_g_: 27.6 Å; homodimeric rabbit enolase, MW: 86.4 kDa, R_g_: 28.3 Å; monomeric transferrin, MW: 76.9 kDa, R_g_: 31.1 Å; and homodimeric BSA, MW: 137 kDa, R_g_: 36.2 Å).

Kratky analysis of the SAXS data also supports that GST-TrbB_WT_ is partially disordered. Deviations from the coordinates (1.732, 1.104) in the Kratky plot (Fig. 4D, marked by red-dashed cross) indicate structural disorder (Da Vela and Svergun, 2020). A partially disordered protein is often indicated by a bell-shaped Gaussian peak that gradually returns to the baseline (Rambo and Tainer, 2013); this is observed in the Kratky plot (Fig. 4D). Accordingly, SAXS bead models are often utilized with high-resolution models, such as that solved by X-ray crystallography or NMR, to determine whether the protein adopts a wider range of conformations that high-resolution methods cannot determine (Kikhney and Svergun, 2015; Powers et al., 2019). TrbB_WT_ has evaded high-resolution structure characterization, but the SAXS (Kratky plot, Fig. 4D), CF-AF2 (Fig. 1B), and ^1^H–^15^N HSQC spectroscopy (Fig. 3, pronounced clustering of TrbB_WT_ [magenta] ^1^H resonances about 8–8.5 ppm; Section 3.1) indicate that TrbB_WT_ is partially disordered (Fig. 1A–C; Supplementary Figs. S1 and S2C), and that the protein is dynamic based on its over-estimated R_g_ values (Fig. 4B and C; Table 1). These insights informed the design of the truncation mutant, TrbB_37-161_. Based on the normalized Pair-distance P(r) distribution function (Fig. 4C), GST-TrbB_WT_ has an elongated shape as opposed to globular (bell-shaped Gaussian), dumbbell (bimodal), or a core-shell (leading asymmetric peak close to the D_max_) (Da Vela and Svergun, 2020). The P(r) curve (Fig. 4C) is observed as a tailing asymmetric peak marked by a modest second peak mid *r*, and gradually approaches 0 at high *r*, suggesting an elongated shape.

EOM was employed to generate models for GST-TrbB_WT_ with the disordered N-terminal TrbB_WT_ treated as a flexible linker. The EOM *χ*^*2*^ = 0.88 (Fig. 4E) indicates that the discrepancy between models from the selected ensemble and empirical SAXS data is low (*χ*^*2*^ → 1 is ideal; Franke et al., 2015; Tossavainen et al., 2018; Tria et al., 2015). The *R*_*flex*_ for the selected ensemble (67.42%, Fig. 4F) indicates a significant degree of conformational polydispersity, supporting the inference of a dynamic GST-TrbB_WT_. The quantitative parameters in Fig. 4F show the lower *R*_*flex*_ of the selected ensemble (*R*_*flex*_ = 67.42%) compared to that of the pool of conformational possibilities (*R*_*flex*_ = 83.63%), indicating a high degree of conformational polydispersity. The ratio of the standard deviation among conformations in the selected ensemble and the standard deviation among conformations in the pool of random possible conformations is described by *R*_*σ*_. Since *R*_*σ*_ = 0.98, the standard deviation of the conformations in the selected ensemble (numerator) is modestly lower compared to that of the pool of possible conformations (denominator), meaning there are more conformations possible than in the selected ensemble modelled in Fig. 4G (Da Vela and Svergun, 2020; Tria et al., 2015). Overall, GST-TrbB_WT_ is highly dynamic due to its high degree of conformational polydispersity.

Fig. 4G diagrammatically summarizes the SAXS/EOM analyses, illustrating the central role of the N-terminus in the chimeric protein's dynamics (Fig. 4G green surface representation). Changes in D_max_ and R_g_ represented by the structural models illustrate GST-TrbB_WT_'s conformational dynamics. Due to the disordered N-terminus (residues S1-R36 according to CF-AF2, Fig. 1A-**C**), GST-TrbB_WT_ is intramolecularly flexible, adopting five representative conformations from a compact and well-folded three-body system (Fig. 4G: Model 1) to a disordered and dispersed system (Fig. 4G: Model 5). The other three structural models (Fig. 4G: Models 2–4) are intermediate conformations that are observed to be less compact compared to Model 1. In considering the fractional contribution of each conformer, GST-TrbB_WT_ appears to adopt conformer 1 at a large probability (Fig. 4G: Model 1, Fraction 5/9), while the other four conformers are each only adopted at a smaller probability (Fig. 4G: Models 1, 3–5, Fraction 1/9).

From a dynamic equilibrium perspective, the greater probability of conformer 1 existing suggests that it is likely the most transiently stable conformer. This model (Fig. 4G: Model 1) indicates that two TrbB_37-161_ molecules are well-packed with each other. CORAL indicates that the point-of-contact between two GST-TrbB_WT_ is through TrbB_37-161_ (χ^2^ = 1.41, Supplementary Fig. S4C), which is supported by the improved EOM fit (χ^2^ = 0.97, Supplementary Fig. S5A; 0.9 ≤ *χ*^*2*^ ≤ 1.1 is ideal; Franke et al., 2015) compared to that which is linked through GST moieties (χ^2^ = 0.88, Supplementary Fig. S5B). This result is likely a phenomenon caused by two factors; firstly, a large proportion of GST-TrbB molecules in solution likely have both GST and TrbB_37-161_ in a well-packed interface such that CORAL and EOM classify them as both forming a dimer, when the two TrbB_37-161_ molecules could simply be within very close proximity at a given moment due to their dynamic folding/unfolding cycles. TrbB's dynamic intramolecular flexibility likely contributes to its function as a DI. The second is that GST may have some structural disorder at its C-terminal when it is part of a chimeric construct with TrbB_WT_ (Kuhnert et al., 2005; Smyth et al., 2003); as both EOM and CORAL are using data and models that fit well with 4 bodies of which two are connected, the result may fit better with a smaller GST model dimerized. Hence, when the TrbB _37-161_ dimer (smaller MW vs. that of GST) is specified as the interface for contact in the CORAL and EOM experimental parameters, a better fit of the output model to the scattering curve is observed. The EOM and CORAL results indicate that in the context of a GST-TrbB dimer, TrbB can adopt a well-packed state transiently, providing some evidence for why untagged TrbB is prone to aggregation and thereby difficult to study *in vitro* using methods that require high protein concentrations, such as X-ray crystallography or SAXS.

When R_g_ values (Fig. 4G) are arranged in an ascending order, the pattern from the D_max_ values surprisingly do not correlate with the R_g_ ascending trend (Supplementary Fig. S6). While R_g_ values have an ascending trend (Model 1 to 5), D_max_ values appear to increase (Model 1 to 3), decrease (Model 3 to 4), then increase (Model 4 to 5); this can be attributed to the difference in dynamics of one locale of the protein (indicated by R_g_) compared to the dynamics of the whole protein (indicated by D_max_), providing additional evidence of intramolecular flexibility. Notably, model 1 shows the most abundant conformation (Fraction: 5/9, Fig. 4G) and TrbB_WT_ (magenta) appears to be in its most compact state compared to other conformers (Model 2 to 5). The four-body system is also generally compact. The significant jumps in D_max_ between conformers (Model 1 to 2, ΔD_max_ = 31.10 Å, a ∼26% increase; Model 2 to 3, ΔD_max_ = 66.70 Å, a ∼44% increase; Model 3 to 4, ΔD_max_ = −48.10 Å, a ∼22% decrease; Model 4 to 5, ΔD_max_ = 61.50 Å, a ∼36% increase) support the flexibility of GST-TrbB_WT_ owing to the N-terminus (Fig. 4G, green) and the fold of TrbB_37-161_ as it interacts with GST (observe the three-body system going from compact to dispersed across the five models, Fig. 4G) and with itself (observe magenta changing conformations across the five models, Fig. 4G). In comparing conformer 1 (Model 1, D_max_ = 119.40 Å, Fig. 4G) and conformer 5 (Model 5, D_max_ = 230.60 Å, Fig. 4G), a 93% increase in D_max_ is observed, explained by the transition from a compact three-body system to a well-dispersed system, and the changes in the observed D_max_ is caused by the changes in the flexibility of the N-terminus (green) and the intramolecular fold of TrbB_37-161_ (magenta).

### 3.3 The disordered N-terminus is not required for disulfide isomerase activity *in vitro*

**Fig. 5:**
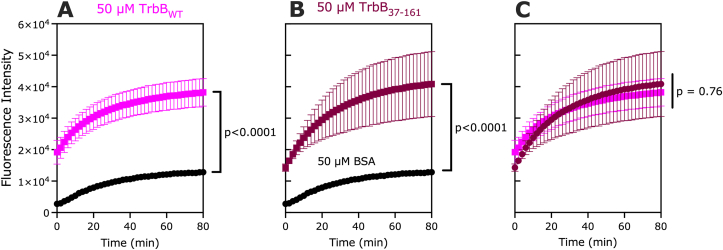
**TrbB**_**37-161**_**functions as a disulfide isomerase *in vitro***. Fluorometric assay at Excitation/Emission = 490nm/580 nm of (A) TrbB_WT_ (magenta) and (B) N-terminal-truncated TrbB_37-161_ (maroon). (C) A statistical *t*-test comparison between the two TrbB constructs. Data points are mean ± SD, n = 6 (two independent samples, each with 3 technical replicates). Bovine Serum Albumin (BSA, black), having no disulfide isomerase activity, serves as a negative control.

The effects of N-terminal deletion on the enzymatic activity of TrbB *in vitro* was investigated. First, we re-established that TrbB_WT_ does function as a disulfide isomerase (DI) *in vitro* compared to Bovine Serum Albumin (BSA), known not to function as a DI, to a statistically significant difference (p < 0.0001) (Fig. 5A; Supplementary Table S1). Second, the truncation of the N-terminal region of TrbB_WT_ (S1-R36, Fig. 1A-**B**) was shown to have no effect on the DI activity of the protein *in vitro* as observed by assaying 50 μM TrbB_37-161_ compared to 50 μM BSA (Fig. 5B; Supplementary Table S1); a statistically significant difference was maintained (p < 0.0001). Comparison between TrbB_WT_ and TrbB_37-161_ indicated no significant difference (p = 0.76) between the two protein constructs (Fig. 5C; Supplementary Table S1). This data is consistent with the CF-AF2 model and previously reported data suggesting a disordered N-terminus of TrbB_WT_ (Hemmis et al., 2011). The data herein suggests that the thioredoxin domain of TrbB is sufficient for DI activity, and the disordered N-terminal region of the protein does not play a role in TrbB's function, supporting previous findings from studies that determined the effect of mutating the active site residues (Elton et al., 2005; Hemmis et al., 2011).

### 3.4 TrbB does not form a stable complex with TraW *in vitro*

**Fig. 6:**
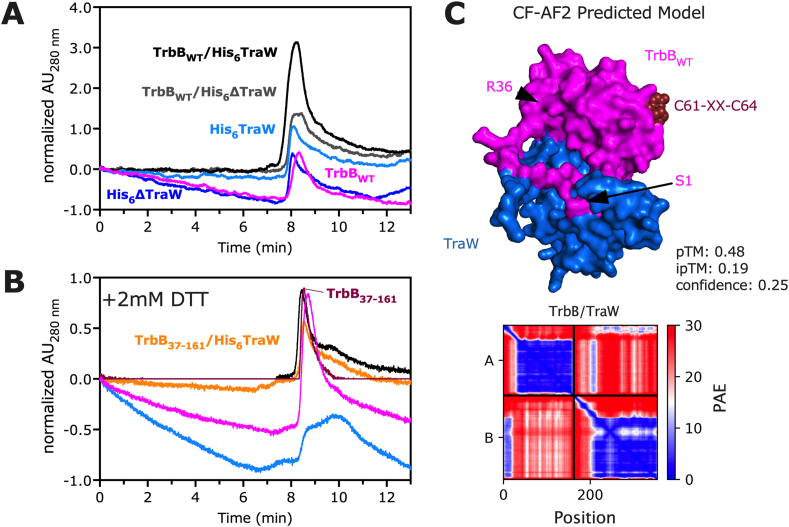
**Chromatographic Investigation of the predicted TrbB/TraW interaction.** (A) Size Exclusion Chromatography elution profiles of TrbB, His_6_TraW, His_6_ΔTraW, TrbB/His_6_TraW, and TrbB/His_6_ΔTraW mixtures. (B) SEC elution profiles of TrbB, TrbB_37-161_, His_6_TraW, and TrbB_37-161_/His_6_TraW mixture in the presence of 2 mM DTT. Protein samples were incubated for 30 min on ice prior to SEC analysis on a pre-equilibrated column at a flow rate of 1.0 mL/min. (C) Structural prediction of the putative TrbB/TraW complex (top) with model accuracy metrics (predicted TM scores, pTM; interface pTM, ipTM), and predicted alignment error (PAE) plot (bottom). The confidence metric, in the range ∈ [0,1], is calculated by 0.8*ipTM + 0.2*pTM, weighing the metric for the reliability of binding interfaces (ipTM) more (Evans et al., 2021).

A previous study by Harris and Silverman (2004) proposed the binding of T4SS_F_ proteins, including TraW and TrbB, using yeast two-hybrid analysis. To test whether TrbB binds TraW as previously reported, TrbB_WT_ and TraW as well as their truncation mutants (TrbB_37-161_ and His_6_ΔTraW) were mixed in solution and allowed to equilibrate on ice for at least 30 min and analyzed using SEC (Fig. 6). Elution profiles of individual proteins (TrbB_WT_, TrbB_37-161_, His_6_TraW, His_6_ΔTraW) were observed as supporting previous sedimentation studies demonstrating that TrbB is monomeric in solution (Hemmis et al., 2011). There is no apparent difference in the elution profiles between the individual proteins and their mixtures (TrbB_WT_/His_6_TraW, TrbB_WT_/His_6_ΔTraW), which all elute at ∼8 min (Fig. 6A), though there is an increase in the magnitude of the peak absorbances when comparing the protein mixture and individual proteins attributable to the increased protein in solution (Fig. 6A).

TrbB is a disulfide isomerase (this study; Hemmis et al., 2011; Elton et al., 2005; Frost et al., 1994), and is involved in the proper formation of disulfide bonds. TraW, with only one cysteine residue, does not have any potential to form any intramolecular disulfide bonds. However, TraW may be a client protein to a disulfide isomerase such as TrbB if it requires guided redox reactions for forming intermolecular cystines. We investigated whether it was important for TrbB to be reduced to bind TraW as TrbB is reported to be active only in its reduced form (Hemmis et al., 2011). In the cell, DsbD is important for the catalytic activity of TrbB; DsbD's role is to reduce TrbB. *In lieu* of DsbD, TrbB was reduced using 2 mM DTT as previously done to assay the activity of DsbC (Chen et al., 1999). However, TrbB, even in its reduced form, does not bind TraW (Fig. 6B).

We modelled the predicted TrbB-TraW complex to further understand the putative binding interaction between TrbB and TraW. The computational model shows that TrbB_WT_ and TraW could indeed interact (Fig. 6C top), although with a high predicted alignment error (PAE) (Fig. 6C bottom), and a low predicted template modelling score (pTM, 0.48), interface pTM (ipTM, 0.19) and confidence (a weighted combination of ipTM and pTM) of 0.25 (Evans et al., 2021). The model predicts that the N-terminal residues of TrbB_WT_ are involved in binding N-terminus of TraW, and that TrbB's CXXC motif in its thioredoxin domain is not located at the binding interface (Fig. 6C). Therefore, while TrbB likely functions as a protein chaperone for other T4SS_F_ proteins (Bragagnolo et al., 2020; Elton et al., 2005; Frost et al., 1994), it does not bind TraW *in vitro*. The difference between our current study (Fig. 6A-**B**) and that of Harris and Silverman (2004) requires further empirical investigations to ascertain whether TrbB binds TraW. For instance, while yeast two-hybrid analysis is a powerful method to identify protein-protein interactions, high intracellular traffic can lead to non-specific binding and the detection of confounding protein-protein interactions, among other limitations (Huang et al., 2007; Rajagopala et al., 2009; Stellberger et al., 2010). Next, we utilized purified proteins in the current study whereas the proteins may require other conditions *in vivo*, such as the presence of the lipid membrane or other microenvironment conditions to interact. Our current *in vitro* binding study employed protein concentrations that may not have been sufficiently high to form an amount of the complex observable in the SEC elution profiles, or even that equilibration times were not sufficient for stable complex formation. Therefore, further studies are on-going to exam the rate at which TrbB binds TraW by an empirical determination of k_on_ (Jarmoskaite et al., 2020) employing orthogonal methods such as Bio-Layer Interferometry to determine the k_on_, k_off_, and K_D_ of the TrbB/TraW interaction (Petersen, 2017). It is also important to consider that chaperones are known as weak and transient binders (Arhar et al., 2021), and that some chaperones have dissociation constant (K_D_) values in the order of 200 μM (Lee et al., 2015). Understanding this principle is guiding our investigations on TrbB's interactions using methods that can probe transient interactions such as Fluorescence Resonance Energy Transfer (Nguyen et al., 2022).

## 4 Conclusions

Disulfide isomerases and other pathways assisting protein folding are critical to ensure proper structural stability and function of proteins. In conjugative T4SSs, like that of the *E. coli* F plasmid, properly folded proteins and their interactions are critical to effectively affect conjugative DNA transfer from donor to recipient cell. There are several T4SS proteins in F-like plasmids that have high cysteine content, and therefore the plasmid encodes for a disulfide isomerase, TrbB, to aid in maintaining proper protein folding. In the current study, we present a solution characterization of the TrbB protein from the F-T4SS, which contains a disordered N-terminal domain and a C-terminal region containing an active thioredoxin motif. Utilizing CD and ^1^H–^15^N HSQC-NMR spectroscopies, we show that removal of the N-terminal region of the protein, the TrbB_37-161_ construct, results in a more thermally stable and well-ordered protein than TrbB_WT_ (Fig. 1, Fig. 2, Fig. 3). Furthermore, removal of the 36 residue N-terminal region of the protein does not affect the disulfide isomerase (DI) activity of the protein *in vitro* (Fig. 5). SEC-MALS-SAXS analysis of GST-TrbB_WT_ (Fig. 4) shows a dynamic and dimeric structure, most likely dimerized via the GST tag, that can adopt multiple conformations in solution largely due to the disordered N-terminal region of TrbB; it is this region that provides full-length TrbB with some of its structurally dynamic nature. A previously identified binding partner, TraW, was not observed to interact with TrbB *in vitro*, although CF-AF2 modelling does suggest that a TrbB-TraW complex is possible, mediated by the N-terminal regions of each protein (Fig. 6). These studies suggest that TrbB employs it's disordered and dynamic N-terminal region as a point of interaction with other T4SS proteins to enable DI activity to ensure proper protein folding, function, and assembly of other F-plasmid proteins to facilitate T4SS_F_-mediated conjugative DNA transfer.

## CRediT authorship contribution statement

**Arnold J. Apostol:** Methodology, Investigation, Validation, Visualization, Writing – Original, Review & Editing. **Nicholas J. Bragagnolo:** Investigation, Visualization, Writing – Original, Review & Editing. **Christina S. Rodriguez:** Visualization, Writing – Original, Review & Editing. **Gerald F. Audette:** Conceptualization, Methodology, Validation, Visualization, Writing – Original, Review & Editing, Supervision, Project administration, Funding acquisition.

## Declaration of competing interest

The authors declare that they have no known competing financial interests or personal relationships that could have appeared to influence the work reported in this paper.

## Data Availability

Data will be made available on request.
