# Supplementary material for: Structural insights into the disulfide isomerase and chaperone activity of TrbB of the F plasmid type IV secretion system
Source: Curr Res Struct Biol. 2024 Jul 14;8:100156. doi: 10.1016/j.crstbi.2024.100156 (PMC11315126; doi:10.1016/j.crstbi.2024.100156)
Supplement: Multimedia component 1 [file mmc1.docx]

**Supplementary Data**

**Structural Insights into the Disulfide Isomerase and Chaperone Activity of TrbB of the F plasmid Type IV Secretion System**

Arnold J. Apostol^1,2^, Nicholas J. Bragagnolo^1,2^, Christina S. Rodriguez^1,2^, Gerald F. Audette^1,2*^

^1^*Department of Chemistry, York University, 4700 Keele St, Toronto, ON, Canada M3J 1P3*

^2^*Centre for Research on Biomolecular Interactions, York University*

**Corresponding Author (audette@yorku.ca)*


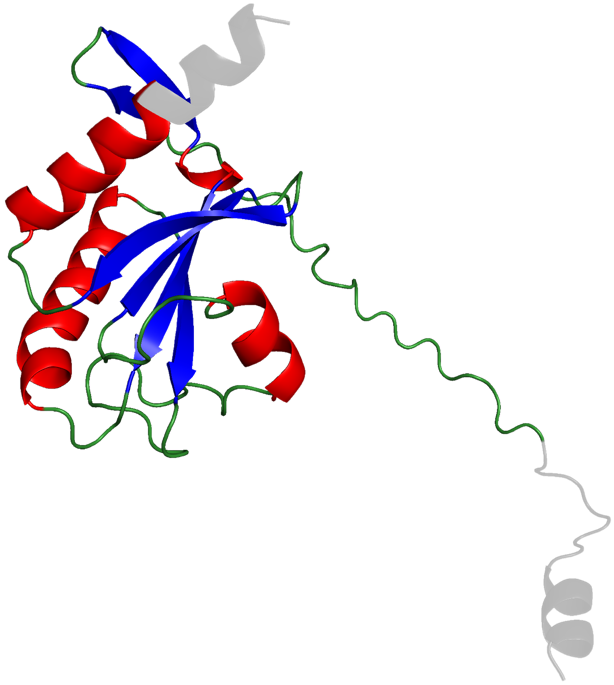


C-terminus

∆T17-R36

in TrbB_37-161_ construct

pTM: 0.74

N-terminus

| Signal sequence (Teufel et al., 2022) | M_1_SLTKSLLFTLLLSAAAVQA_20_ |
| --- | --- |
| TrbB_WT_ sequence (∆signal sequence) | **S_1_TRDEIERLWNPQGMATQPAQPAAGTSARTAKPAPRWFRLSNGRQVNLADWKVVLFMQGHCPYCHQFDPVLKQLAQQYGFSVFSYTLDGQGDTAFPEALPVPPDVMQTFFPNIPVATPTTFLVNVNTLEALPLLQGATDAAGFMARVDTVLQMYGGKKGAK_161_** |
| Thioredoxin domain (TD) | **T_17_QPAQPAAGTSARTAKPAPRWFRLSNGRQVNLADWKVVLFMQGHCPYCHQFDPVLKQLAQQYGFSVFSYTLDGQGDTAFPEALPVPPDVMQTFFPNIPVATPTTFLVNVNTLEALPLLQGATDAAGFMARVDTVLQ_152_** |
| Residues of TD deleted in TrbB_37-161_ | **T_17_QPAQPAAGTSARTAKPAPR_36_** |

**Supplementary Figure S1. ColabFold-AlphaFold2 model for TrbB_WT_ emphasizing the thioredoxin domain and residues T37-R56 deleted in TrbB_37-161_ construct.** (A) The full amino acid sequence, the signal sequence (predicted by SignalP; Teufel at al., 2022) that gets cleaved to form the mature protein *in vivo*, the thioredoxin domain (Uniprot: P18035; Prosite: PRU00691), and the residues deleted in TrbB_37-161_ (dotted circle) are shown. Residues are coloured based on CF-AF2’s predicted secondary structures (Mirdita et al., 2022); red for α-helices, blue for β-sheets, and green for loops. Only the thioredoxin domain is shown in colour.

**A**

TrbB_37-161_ amino acid sequence

**WFRLSNGRQVNLADWKVVLFMQGHCPYCHQFDPVLKQLAQQYGFSVFSYTLDGQGDTAFPEALPVPPDVMQTFFPNIPVATPTTFLVNVNTLEALPLLQGATDAAGFMARVDTVLQMYGGKKGAK**

**B**

TrbB_37-161_ amino acids forming α-helices

|  | **residues** | **No. residues** | **%** |
| --- | --- | --- | --- |
| -helical residues | **LADPYCHQFDPVLKQLAQQYPDVMQTFAAGFMARVDTVLQM**  **YGGKKGA** | 48 | 38.4 |
| polar uncharged | **YCQQQQYQTTQY** | 12 | 25.0 |
| negatively charged* | **DDDD** | 4 | 8.3 |
| positively charged* | **HKRKK** | 5 | 10.4 |
| polar & charged |  | 21 | 43.8 |

*at physiological pH

**C**

CF-AF2 model for TrbB_37-161_ in 2 orientations


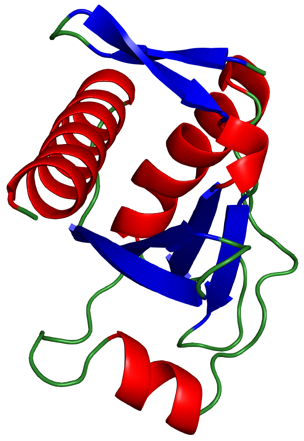


N-terminus

C-terminus


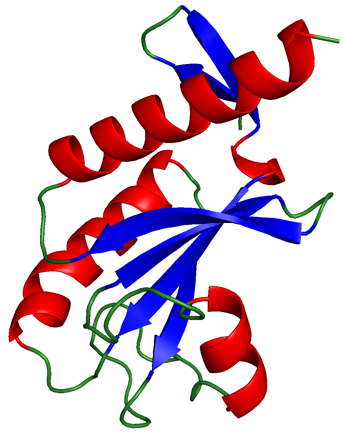


N-terminus

C-terminus

pTM: 0.88

**Supplementary Figure S2. TrbB_37-161_ residues predicted to form α-helices.** (A) The full amino acid sequence, (B) amino acids predicted by ColabFold-AlphaFold2 (CF-AF2) to form α-helices (in red; in table), and the amino acids classified according to their polarity and charge (table) are shown. (C) CF-AF2 3D structure is shown to provide some insights into the organization of the secondary structures in the protein in 3D space; left model distinguishably shows the N- and C-terminus of the protein. Residues in blue are β-sheets, and those in green are loops. CF-AF2 is described by Mirdita et al. (2022). Predicted structure models were visualized in PyMOL v2.5.2 (Schrödinger Inc.).

~86 kDa

| **Method** | **Estimated molecular weight (kDa)** |
| --- | --- |
| amino acid sequence (Gasteiger et al., 2003)* | 46.4 (monomer); 92.8 (homodimer) |
| Porod Volume (Vp; Piiadov et al., 2019) | 119.6 |
| Volume of correlation (Vc; Rambo and Tainer, 2013) | 102.6 |
| Shape & Size (Franke et al., 2018) | 106.4 |
| Bayes (Hajizadeh et al., 2018) | 109.1 |

*Determined computationally by Expasy ProtParam; other methods are experimental based on SAXS

**Supplementary Figure S3. Size Exclusion Chromatography (SEC) Multi Angle Light Scattering (MALS) on protein sample and molecular weight estimations characteristic of a homodimeric GST-TrbB.** SEC-MALS was collected by the BioCAT 18ID beamline facility (Advanced Photon Source, Argonne National Laboratory, USA) using a GE Superdex 200 Increase 10/300 column at a flow rate of 0.6 mL/min coupled to a Wyatt DAWN Heleos II MALS system. Molecular weight estimations were based on SAXS data determined using BioXTAS RAW v.2.2.1 (Hopkins et al., 2017).

**
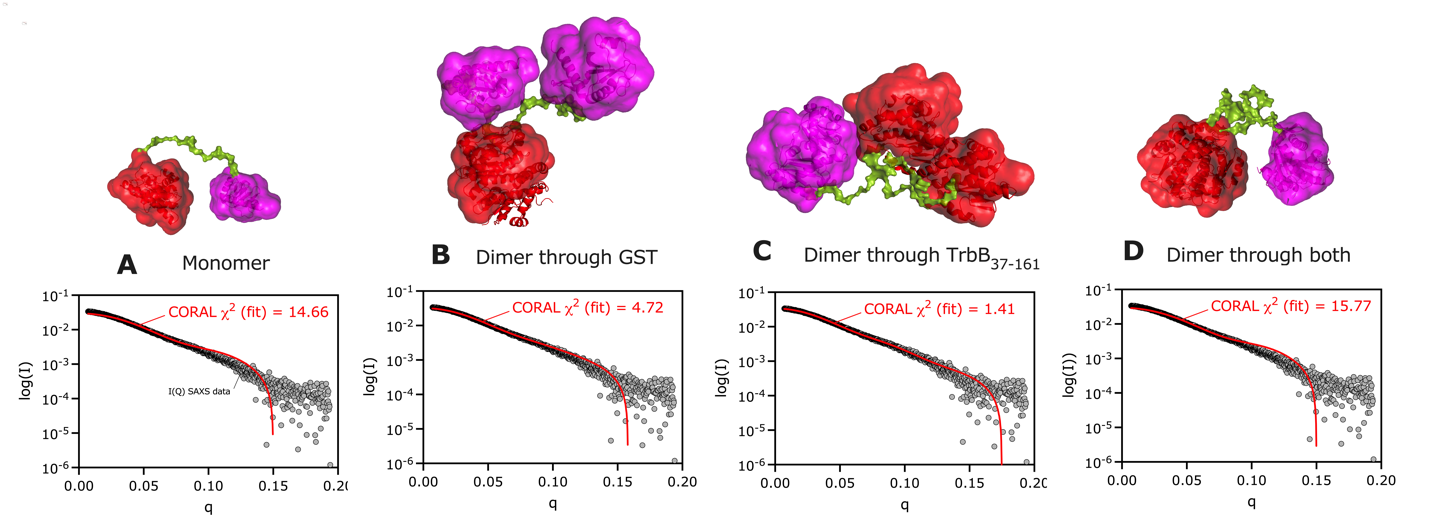
Supplementary Figure S4. GST-TrbB_WT_ is a homodimer according to CORAL.** The SAXS scattering data was analyzed using the CORAL approach to determine whether the data better fits a (**A**) monomeric or (**B-D**) dimeric construct.

**
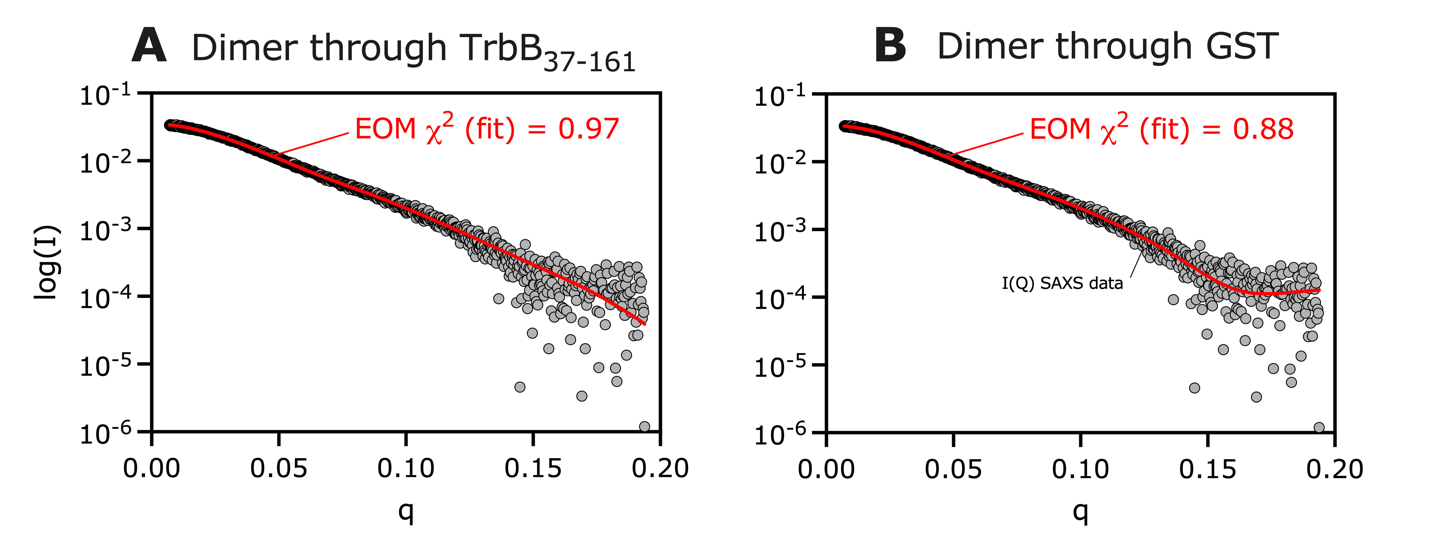
Supplementary Figure S5. Homodimeric GST-TrbB_WT_ points of contact according to EOM.** The SAXS scattering data was analyzed using the EOM approach to further explore whether homodimeric GST-TrbB_WT_ interacts through (**A**) TrbB_37-161_ moities or (**B**) GST.

| **Supplementary** **Table S1. Welch's T-test for Figure 5.** Analysis was performed using GraphPad Prism v.9.5.1 for macOS. Bovine Serum Albumin (BSA) is a negative control. | | | |
| --- | --- | --- | --- |
|  | **TrbB_WT_ vs. BSA** | **TrbB_37-161_ vs. BSA** | **TrbB_WT_ vs. TrbB_37-161_** |
| Column B | 50 µM TrbB_WT_ | 50 µM TrbB_37-161_ | 50 µM TrbB_WT_ |
| vs. | vs. | vs. | vs. |
| Column A | 50 µM BSA | 50 µM BSA | 50 µM TrbB_37-161_ |
| **Unpaired t test with Welch's correction** | | | |
| P value | <0.0001 | <0.0001 | 0.7633 |
| P value summary | **** | **** | ns |
| Significantly different (P < 0.05)? | Yes | Yes | No |
| One- or two-tailed P value? | Two-tailed | Two-tailed | Two-tailed |
| Welch-corrected t, df | t=24.97, df=64.83 | t=19.13, df=53.29 | t=0.3023, df=71.67 |
| **How big is the difference?** | | | |
| Mean of column A | 9662 | 9662 | 33329 |
| Mean of column B | 32907 | 33329 | 32907 |
| Difference between means (B - A) ± SEM | 23245 ± 930.8 | 23667 ± 1237 | -422.2 ± 1397 |
| 95% confidence interval | 21386 to 25104 | 21185 to 26149 | -3206 to 2362 |
| R squared (eta squared) | 0.9058 | 0.8728 | 0.001274 |
| **F test to compare variances** | | | |
| F, DFn, Dfd | 2.874, 40, 40 | 5.847, 40, 40 | 2.035, 40, 40 |
| P value | 0.0012 | <0.0001 | 0.0272 |
| P value summary | ** | **** | * |
| Significantly different (P < 0.05)? | Yes | Yes | Yes |
| **Data analyzed** | | | |
| Sample size, column A | 41 | 41 | 41 |
| Sample size, column B | 41 | 41 | 41 |

**
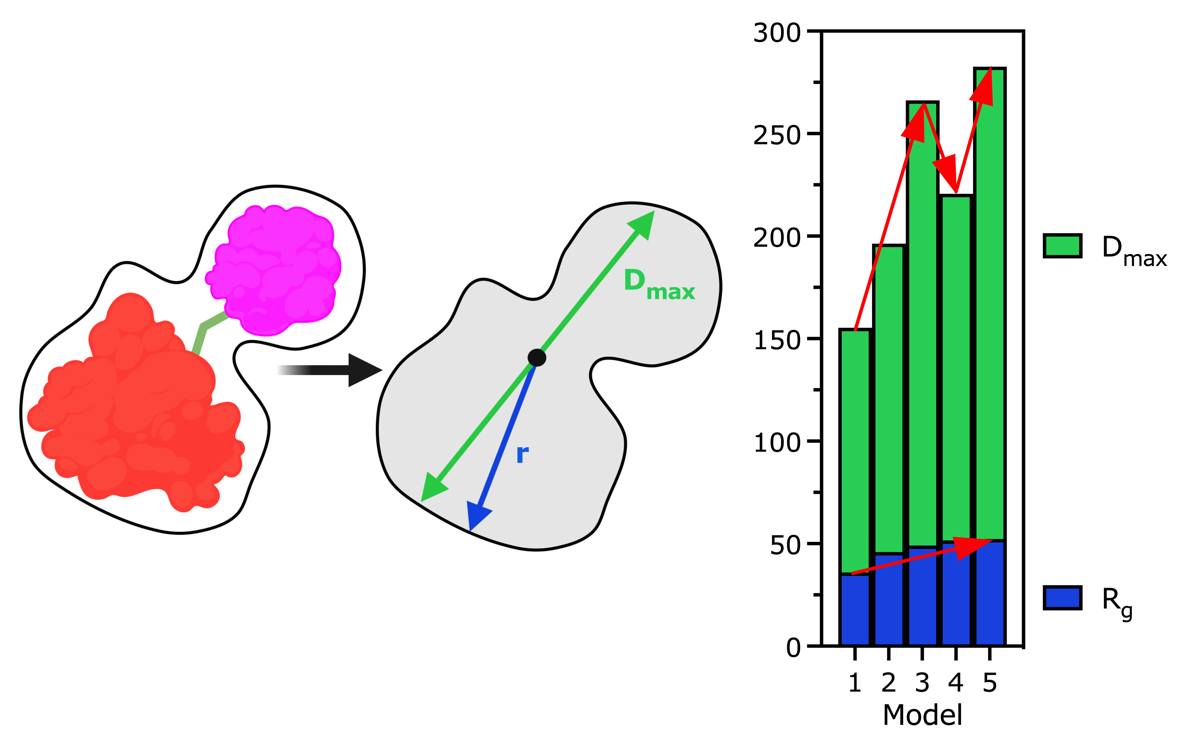
Supplementary Figure S6. The non-correlational pattern of R_g_ and D_max_ across GST-TrbB_WT_ SAXS-EOM models indicates the protein’s intramolecular dynamicity.** Diagrammatic representation of SAXS-derived shape of GST-TrbB_WT_ (left), with the D_max_ and R_g_ (weighted root mean square of the intramolecular distances [*r*] with respect to the centroid of the electron density) represented (middle). Histogram (right) was derived from the EOM parameters shown in Figure 4G.

**References**

Franke, D., Jeffries, C.M., Svergun, D.I., 2018. Machine Learning Methods for X-Ray Scattering Data Analysis from Biomacromolecular Solutions. Biophys J 114, 2485–2492. https://doi.org/https://doi.org/10.1016/j.bpj.2018.04.018

Gasteiger, E., Gattiker, A., Hoogland, C., Ivanyi, I., Appel, R.D., Bairoch, A., 2003. ExPASy: the proteomics server for in-depth protein knowledge and analysis. Nucleic Acids Res 31, 3784–3788. https://doi.org/10.1093/nar/gkg563

Hajizadeh, N.R., Franke, D., Jeffries, C.M., Svergun, D.I., 2018. Consensus Bayesian assessment of protein molecular mass from solution X-ray scattering data. Sci Rep 8, 7204. https://doi.org/10.1038/s41598-018-25355-2

Hopkins, J.B., Gillilan, R.E., Skou, S., 2017. BioXTAS RAW: Improvements to a free open-source program for small-angle X-ray scattering data reduction and analysis. J Appl Crystallogr 50, 1545–1553. https://doi.org/10.1107/S1600576717011438

Mirdita, M., Schütze, K., Moriwaki, Y., Heo, L., Ovchinnikov, S., Steinegger, M., 2022. ColabFold: making protein folding accessible to all. Nat Methods 19, 679–682. https://doi.org/10.1038/s41592-022-01488-1

Piiadov, V., Ares de Araújo, E., Oliveira Neto, M., Craievich, A.F., Polikarpov, I., 2019. SAXSMoW 2.0: Online calculator of the molecular weight of proteins in dilute solution from experimental SAXS data measured on a relative scale. Protein Science 28, 454–463. https://doi.org/https://doi.org/10.1002/pro.3528

Rambo, R.P., Tainer, J.A., 2013. Accurate assessment of mass, models and resolution by small-angle scattering. Nature 496, 477–481. https://doi.org/10.1038/nature12070

Teufel, F., Almagro Armenteros, J.J., Johansen, A.R., Gíslason, M.H., Pihl, S.I., Tsirigos, K.D., Winther, O., Brunak, S., von Heijne, G., Nielsen, H., 2022. SignalP 6.0 predicts all five types of signal peptides using protein language models. Nat Biotechnol 40, 1023–1025. https://doi.org/10.1038/s41587-021-01156-3
